# Influence of Silane Treatment on CNM/PAC/PVDF Properties and Performance for Water Desalination by VMD

**DOI:** 10.3390/membranes15040104

**Published:** 2025-04-01

**Authors:** Samraa R. Khaleel, Salah S. Ibrahim, Alessandra Criscuoli, Alberto Figoli, Dahiru U. Lawal, Qusay F. Alsalhy

**Affiliations:** 1 Membrane Technology Research Unit, Chemical Engineering Department, University of Technology-Iraq, Alsinaa Street 52, Baghdad 10066, Iraq; che.21.02@grad.uotechnology.edu.iq (S.R.K.); salah.s.ibrahim@uotechnology.edu.iq (S.S.I.); 2 Institute on Membrane Technology (CNR-ITM), Via P. Bucci 17/C, 87036 Rende, CS, Italy; a.figoli@itm.cnr.it; 3 Interdisciplinary Research Center for Membrane and Water Security, King Fahd University of Petroleum and Minerals, Dhahran 31261, Saudi Arabia; dahiru.lawal@kfupm.edu.sa; 4 Mechanical Engineering Department, King Fahd University of Petroleum and Minerals, Dhahran 31261, Saudi Arabia

**Keywords:** PFTES, vacuum membrane distillation, membrane, coating layer, desalination, carbon nanomaterials

## Abstract

Vacuum membrane distillation (VMD) is a promising process for water desalination. However, it suffers some obstacles, such as fouling and wetting, due to the inadequate hydrophobicity of the membrane and high vacuum pressure on the permeate side. Therefore, improving surface hydrophobicity and roughness is important. In this study, the effect of 1H,1H,2H,2H-Perfluorodecyltriethoxysilane (PFTES) on the morphology and performance of CNM/PAC/PVDF membranes at various concentrations was investigated for the first time. Membrane characteristics such as FTIR, XRD, FE-SEM, EDX, contact angle, and hydrophobicity before and after modification were analyzed and tested using VMD for water desalination. The results showed that the membrane coated with 1 wt.% PFTES had a higher permeate flux and lower rejection than the membranes coated with the 2 wt.% PFTES. The 2 wt.% PFTES enhanced the contact angle to 117° and increased the salt rejection above 99.9%, with the permeate flux set to 23.2 L/m^2^·h and at a 35 g/L NaCl feed solution, 65 °C feed temperature, a 0.6 L/min feed flow rate, and 21 kPa (abs) vacuum pressure. This means that 2 wt.% PFTES-coated PVDF membranes exhibited slightly lower permeate flux with higher hydrophobicity, salt rejection, and stability over long-term operation. These outstanding results indicate the potential of the novel CNM/PAC/PVDF/PFTES membranes for saline water desalination. Moreover, this study presents useful guidance for the enhancement of membrane structures and physical properties in the field of saline water desalination using porous CNM/PAC/PVDF/PFTES membranes.

## 1. Introduction

Over the past few decades, freshwater availability has been under high stress due to climate change, growing industrialization, and population [1,2]. Globally, more than two billion people do not have access to drinking water, and by 2050, it is anticipated that it will be scarce for fifty percent of the world’s population. Desalination technologies can help increase the supply of fresh water by recovering water from contaminated or salty water, such as brackish water, seawater, and industrial wastewater [3]. Membrane distillation is a promising technique for treating high-salt water and wastewater [4,5,6]. MD is a non-isothermal process that operates based on the vapor pressure difference between the feed and permeate sides of the microporous hydrophobic membrane [7,8,9].

The literature mostly concerns four main configurations of MD: direct contact membrane distillation (DCMD), air gap membrane distillation (AGMD), sweep gas membrane distillation (SGMD), and vacuum membrane distillation (VMD), depending on the way water accumulates on the permeate side. Compared with other desalination techniques, VMD has attracted more attention due to the low heat loss by conduction and higher permeate flux [10,11,12,13]. Despite these advantages, it has a low resistance to pore wetting and fouling [14,15]. For ideal rejection and permeability in MD, a hydrophobic microporous membrane is the most important property of MD membrane, which is fabricated from hydrophobic polymers, such as polytetrafluoroethylene (PTFE), polypropylene (PP), and polyvinylidene fluoride (PVDF). It has low surface-free energy and high water repellency [16,17,18]. More research has been developed to improve the wetting resistance by increasing the surface hydrophobicity, and scientific researchers have noticed that decreasing surface energy and increasing roughness are the main ways to enhance the surface hydrophobicity by surface fluorination [17].

Silane is an effective agent for low surface-free energy materials with hydrophobic ends, which makes it a more suitable monomer for hydrophobic surfaces [19]. It is used for modifying different types of material surfaces by incorporating fluorinated groups on the membrane surface, resulting in reduced pore wetting and enhanced hydrophobicity, salt rejection, stability flux, and fouling resistance [20]. Fluoro-silane has a small atomic radius that attempts to lower surface energy on the outer surface by forming a covalent bond that is stable with carbon. Furthermore, non-polar bonds of carbon–silicon cause reduced surface energy and enhanced hydrophobicity [21]. The long reaction time and low concentration of silane solution make it efficient for the surface modification method [22,23].

In this study, high hydrophobic carbon nanomaterial powder-activated carbon (CNM/PAC) was embedded using different amounts of PVDF polymer. The optimum membrane was modified chemically using different concentrations of Perfluorodecyltriethoxysilane (PFTES) for the first time to enhance the hydrophobicity of the membrane and the salt rejection. The membranes were characterized using several techniques and tested in vacuum membrane distillation (VMD) for the desalination of highly saline water (35 g/L). The effect of operating parameters on the permeate flux of the VMD system was also evaluated.

## 2. Materials and Methods

### 2.1. Chemicals Materials

Polyvinylidene fluoride (PVDF) with a molecular weight of 500–600 g/mol was acquired from Hebei luoxing Tech Co., Ltd., Shijiazhuang, China. N-methyl-2-pyrrolidone (NMP) was purchased from the Chinese company Shandong Zhishang Chemical Co., Ltd., Jinan, China, CNM/PAC high-hydrophobic nanomaterials were synthesized via chemical vapor deposition [24]. 1H,1H,2H,2H-Perfluorodecyltriethoxysilane (PFTES) (C_13_H_13_F_17_O_3_Si at 98% purity and 568.3 g/mol molecular weight was purchased from Jinan Future Chemical Co., Ltd., Jinan, China. N-hexane (C_6_H_14_) with a molecular weight of 86.18 g/mol), sodium chloride (NaCl), and isopropanol with a density of ρ = 0.786 g·cm^−3^ were purchased from Loba company (Bronx, NY, USA).

### 2.2. Membrane Preparation

PVDF flat sheet membranes were prepared using the phase inversion method with varying concentrations of CNM/PAC. Firstly, PVDF powder was dried at 50 °C for 2–3 h to remove any moisture content before fabricating the casting solution. Various amounts of CNM/PAC (0.1, 0.2, 0.3, 0.4, and 0.5%) were separately loaded in NMP solvent. To facilitate dispersion, the mixtures (CNM/PAC and NMP) were sonicated ultrasonically for 30 min in a water bath (24 kHz, Fyang, Fuyang, China). After sonication, PVDF powder at 14% was added gradually to the mixture (CNM/PAC/NMP solution), then stirred magnetically (hot plate, Joan lab, Huzhou, China) at 100–150 rpm and 60 °C for 24 h to achieve a homogenous mixture. Then, it was stored at 50 °C for 2–3 h to remove any trapped air bubbles before casting.

Following that, the dope solution was cast onto a clean glass plate using a film applicator (AFA-IV, Yantai, China) with a 0.25 mm gap of clearance. The membrane casting was immediately immersed in a water bath as a coagulation at room temperature. After some time, the membrane was separated from the glass plate and transferred to a distilled water bath, where it remained for at least 24 h. It was then replaced with other distilled water to ensure the removal of any residual solvent completely. Finally, the prepared membranes were stored in a container with distilled water for further analysis. Details of the dope solution composition are provided in Table 1.

The prepared CNM/PAC/PVDF membranes are chemically modified using a fluorosilane solution, specifically, 1H,1H,2H,2H-Perfluorodecyltrimethoxysilane (PFTES, C_13_H_13_F_17_O_3_Si) by the dip-coating method. To obtain the coating solution, PFTES was dissolved in hexane at two different concentrations (1 and 2 vol./vol.%). Membranes were dip-coated in the PFTES solution for 24 h to obtain sufficient coverage of the surface and enhance the hydrophobicity. After this treatment, the membranes were removed and rinsed several times with distilled water to remove any unreacted material. They were then dried at ambient temperature for 2 h before further analysis

## 3. Membrane Characterization

The chemical composition of the membrane was characterized before and after a long time of operation in VMD. The Attenuated total reflectance Fourier-Transform Infrared Spectroscopy (ATR-FTIR) instrument (IRT-5200 FT-IR, JASCO, Tokyo, Japan) was used to analyze the functional group of the prepared membrane before and after the VMD experiments and to investigate the possible interaction between the membrane material and the components of the feed solution. Also, X-ray diffraction (XRD, Shimadzu, Kyoto, Japan) was used to display the composition of the membranes and the salt deposition after VMD experiments.

The thickness of the membrane was measured by a micrometer (HDT, Beijing, China) with an accuracy of 0.001 mm and estimated by SEM for more accuracy. The value of the average thickness was calculated for five measurements taken at various locations on the sample of the membrane [25]. The porosity of the membrane is defined as the ratio of pore volume to total membrane volume, which is determined by a gravimetric method dependent on the density of the prepared membrane to polymer density according to Equation (1) [26,27].

(1) $$\varepsilon \left(\%\right)=\frac{\left(W_{w}-W_{d}\right)/\rho_{ISO}}{\left(W_{w}-W_{d}\right)/\rho_{ISO}+(W_{d}/\rho_{m})}$$

where $\rho_{m}$ and $\rho_{pISO}$ are the membrane density and isopropanol density (0.786 g/cm^3^), and $W_{w}$ and $W_{d}$ are the weight and dry membrane

(2) $$\rho_{m}=\frac{W_{m}}{Vm}$$

where $W_{m}$ = weight of the membrane (g), Vm = membrane volume (Vm=A × l) A = membrane area (cm^2^), and l is the thickness of the membrane (cm).

The mean pore radius is calculated based on the porosity and permeate flux according to the Guerout–Elford–Ferry Equation (3):

(3) $$r_{m}=\sqrt{\frac{\left(2.9-1.75\varepsilon \right)8\eta lJ}{\varepsilon A\Delta p}}$$

where $r_{m}$ is the mean pore size; η is the viscosity of the water 1 × 10^−3^ (at 65 °C); l is the membrane thickness (m); J is the water permeate flux (L/m^2^·h); ε is the porosity of the membrane; A is the membrane effective area (m^2^); and Δp is the transmembrane pressure (Pa) [23].

The wettability of all the membranes prior and after the VMD experiment is evaluated by the measurement water contact angle (WCA) using Theta Lite-TL100 and TL101- Finland. The WCA will instantaneously indicate the hydrophobic nature of the prepared membrane. A 3 μL sessile droplet of distilled water was applied carefully to the surface of the flat membrane using a synergy, then the WCA between the membrane surface and the drop of water was evaluated. Five readings were measured at various locations for each sample of the membrane to eliminate errors, then the average contact angle was determined at room temperature.

The liquid entry pressure (LEP), or pore entry pressure, is the maximum pressure at which water does not pass through the pores of the membrane. LEP is affected by pore geometry (size, shape) and hydrophobicity [28,29]. The LEP is calculated to evaluate membrane stability over long-term MD applications, using the Laplace (Cantor) Equation (4) [30,31,32]:

(4) $$LEP=P_{f}-P_{p}=\frac{-2\;\beta \;\gamma \;cos\theta }{r_{max}}$$

where $P_{f}\;and\;P_{p}$ represent the feed and permeate sides’ hydraulic pressures of the system, β is the pore coefficient geometric (equal to 1 for cylindrical pores and 0 < β < 1 for non-cylindrical pores), γ is the surface tension of the liquid surface tension, θ is the contact angle on the surface of the membrane, and r_max_ is the maximum pore radius.

The mechanical properties of the prepared membrane were evaluated using a tensile strength testing machine (Tinius Olsen H50KT, England) equipped with computer software for data analysis. The samples of the membrane, measuring 2 cm × 10 cm, were clamped at both ends of the machine and subjected to tension at an elongation speed of 5 mm/min. The load cell had a force of 5 N, and the tests were conducted at room temperature. The tensile strength of each sample was calculated by dividing the force at the point of fracture (N) by the cross-sectional area of the membrane (m^2^) [33].

The morphology and the elemental composition of the membrane surface were tested before and after the VMD experiments by Field Emission Scanning Electron Microscopy (FE-SEM, Zeiss sigma- Germany) combined with Energy Dispersive X-ray spectroscopy analysis (EDS). For cross-section imaging, the samples of the membrane were dried at room temperature for 48 h before being fractured by exposure to the liquid nitrogen, and then the surface and cross-section of the membrane were subsequently coated with a uniform thin layer of gold.

## 4. Membrane Performance

**Figure 1:**
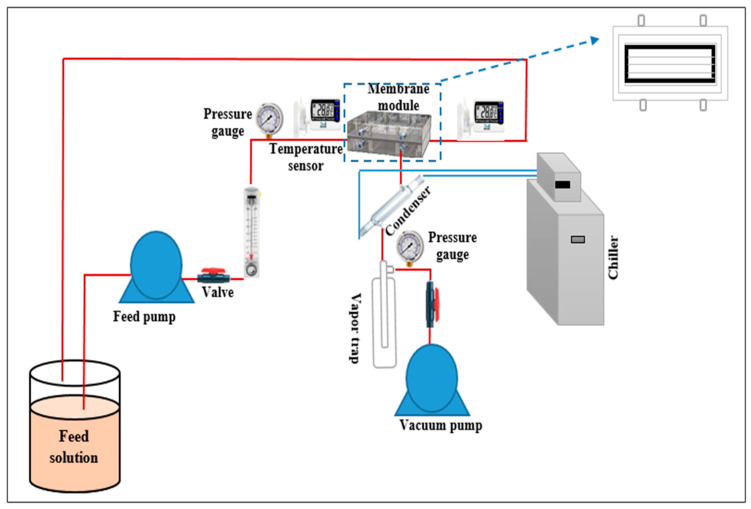
Schematic diagram of VMD system.

The performance of all the prepared membranes was evaluated using a vacuum membrane distillation (VMD) system shown in Figure 1. The permeate flux in VMD was determined using Equation (5) [33,34]:

(5) $$J=\frac{Q}{A\times t}$$

where J represents the permeate flux (L/m^2^·h); Q is the volume of vapor collected (L); A is the effective membrane area (m^2^) calculated as width (W) times length (L); t is the time over which the vapor is collected (h); and the salt rejection (%R) was determined using Equation (6) [29,31]:

(6) $$\%R=(1-\frac{C_{p}}{C_{f}})\times 100$$

where C_f_ and C_p_ represent the feed and permeate concentrations, respectively. To ensure the stability of nanomaterials over a long time of VMD operation, a leaching ratio experiment was performed. Membrane samples (3 × 5 cm) were first dried at 55–60 °C for two hours, weighed, and then immersed in distilled water for one month, with regular water changes. Following the soaking time, the membranes were weighed and dried once more in an oven. The following Equation (7) was used for calculating the leaching ratio [35]:

(7) $$Leaching\;ratio\left(\%\right)=\frac{M_{1}-M_{2}}{M_{1}}\times 100\%$$

where M_1_ and M_2_ represent the membranes’ relative weights prior to and following soaking.

## 5. Results and Discussion

### 5.1. FTIR Analysis

**Figure 2:**
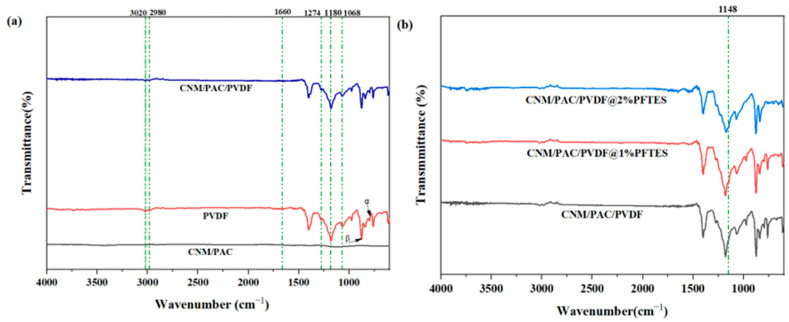
FTIR spectra for (**a**) CNM/PAC, P0, and P4 membranes; (**b**) spectra of control P4 and P4 membranes treated with 1 and 2 wt.% PFTES.

The FT-IR spectra were used to analyze changes in the structure of PVDF membranes after modification with nanomaterials and chemical modifications, as displayed in Figure 2. The peak at 1068 cm^−1^ was related to CH_2_ groups, while the peaks at 1180 cm^−1^ and 1274 cm^−1^ were attributed to carbon–fluorine (C-F) vibration bonds. In addition, the α-phase characteristic is found at 782 cm^−1^, which means the phase is crystalline. The β-phase characteristic peaks were seen at 875 cm^−1^, which means the CF_2_ is stretching in a symmetric way [36]. Also, the 1660 peaks cm⁻¹ were linked to the carbonyl (C-O) group [37], and the bands at 2980 cm^−1^ and 3020 cm^−1^ were linked to the –CH and CH_2_ stretching vibrations [38]. When we compared the pure PVDF with the modified matrix carbon nanomaterial powder-activated carbon (CNM/PAC), we observed weaker peaks at 1274 cm^−1^, 1180 cm^−1^, and 1040 cm^−1^ for the symmetrical and asymmetrical C-F stretching bands, as well as the C-H vibrations peak Figure 2a. These peaks indicate a change in the crystalline structure of polymers after the addition of CNM/PAC.

Figure 2b displays the FT-IR spectra of the CNM/PAC/PVDF membranes before and after chemical modification by the dip-coating method with 1–2 wt.% PFTES for 24 h. As can be seen from that figure, the chemically modified membrane showed a peak at 1148 cm^−1^, which indicated a successfully coated membrane surface with silane solution and Si-O stretching vibrations. Moreover, the membranes coated with 2 wt.% PFTES had a strong Si-O absorption peak and improved the spectral features of fluorine compounds, which confirms efficient modification with PFTES. These results highlight the transformation of the PVDF structure by being embedded with CNM/PAC and coated with PFTES, displaying improved chemical features for enhanced hydrophobicity and membrane performance.

### 5.2. X-Ray Diffraction (XRD)

**Figure 3:**
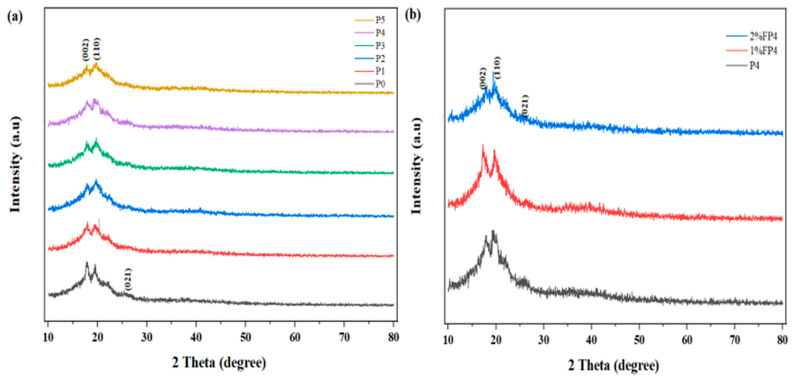
XRD spectra of (**a**) P0, P1, P2, P3, P4, and P5 membranes; (**b**) P4 before and after being chemically modified with 1–2% PFTES (1% FP4) and (2 wt.% FP4) membranes.

The XRD analysis was utilized to determine the crystal phase for the pristine PVDF membrane before and after modification with various concentrations of CNM/PAC membranes, as shown in Figure 3. It can be seen that peaks at 18.2°, 20.32°, and 26.04° are related to α and β phases, respectively [39]. After the addition of CNM/PAC, the intensity of peaks 18.2° (002), 20.23° (110), and 26.04° (021) reduced slightly compared with the crystal structure of the pristine PVDF, as shown in Figure 3a. The intensity of the peak was enhanced after chemically modifying the membrane with 1–2 wt.% PFTES, as seen in Figure 3b.

### 5.3. Field Emission Scanning Electron Microscopy and Energy Dispersive X-Ray Spectrometer (FE-SEM and EDX)

**Figure 4:**
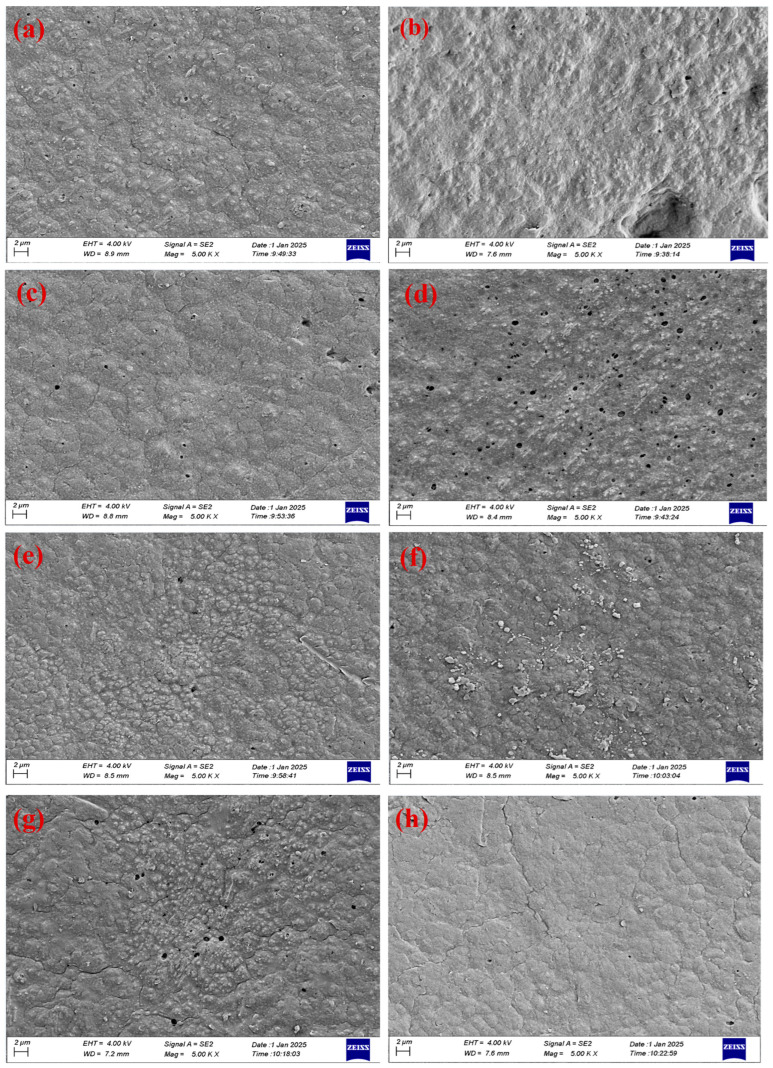
FE-SEM surface image of various concentrations of CNM/PAC/PVDF membranes (**a**) P0 (0%), (**b**) P1 (0.1 wt.%), (**c**) P2 (0.2 wt.%), (**d**) P3 (0.3 wt.%), (**e**) P4 (0.4 wt.%), (**f**) P5 (0.5 wt.%), (**g**) 1 wt.% FP4, (**h**) 2% FP4 membranes.

The morphology changes of the PVDF membrane before and after modification with CNM/PAC nanomaterials and chemical modification with PFTES were analyzed by FE-SEM. In this study, 0.1 wt.% (P1), 0.2 wt.% (P2), 0.3 wt.% (P3), 0.4 wt.% (P4), and 0.5 wt.% (P5) of CNM/PAC were embedded with PVDF by phase inversion. The FE-SEM images of the membrane surface of all the prepared membranes are shown in Figure 4a–f. The pore size increased as the CNM/PAC increased from 0.1 wt.% to 0.4 wt.%. Upon loading 0.5 wt.%, the pore size on the membrane surface decreased, as seen in Figure 4b–f. The change in the structure of the membrane surface after being embedded with CNM/PAC is related to a delay in the rate of exchange between the solvent (NMP) and the non-solvent (water) through the membrane [35,40]. Also, Figure 4g,h shows the FE-SEM images for the top surface of the 0.4 wt.% CNM/PAC/PVDF membrane when treated with 1–2 wt.% PFTES. When the membrane surface was coated with 1% PFTES, the pore size of the membrane surface increased slightly, and no effect after coating with 1% PFTES was observed due to the small molecule structure and concentration of PFTES, whereas the coating with 2 wt.% PFTES resulted in a slight decrease in pore size. This decrease in pore size may be attributed to the narrowed pore size on the surface of the chemically modified membrane, which would lead to a reduction in the permeate flux [17,41].

It was observed that the P3 membrane and 1FP4 membrane surfaces show large amounts of big holes, while other membranes show much fewer holes. This observation was attributed to the increment in the content of CNM/PAC in the casting solution. 1FP4 shows a large pore surface due to the coated P4, with a low concentration of PFTES caused by the evaporation of a hexane-silane solution, which leads to an increase in the surface pore size and porosity.

**Figure 5:**
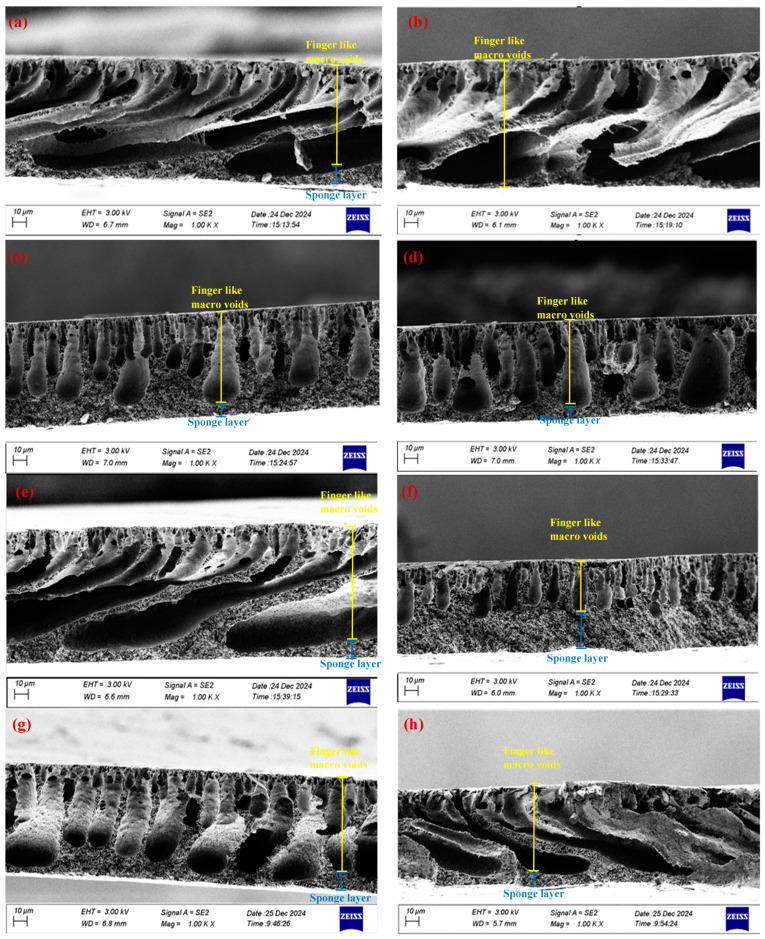
FE-SEM cross-section image of various concentrations of CNM/PAC/PVDF membranes (**a**) P0 (0 wt.%), (**b**) P1 (0.1 wt.%), (**c**) P2 (0.2 wt.%), (**d**) P3 (0.3 wt.%), (**e**) P4 (0.4 wt.%), (**f**) P5 (0.5 wt.%), (**g**) (1 wt.% FP4), (**h**) (2 wt.% FP4) membranes.

The cross-sectional images of the PVDF membranes before and after being modified with different concentrations of CNM/PAC and compared with the chemically modified PVDF are shown in Figure 5. The images show a typical construction with two layers: finger-like macro-voids near the upper surface and sponge-like structures towards the bottom. After loading the nanomaterials, both the size and the number of these macro-voids increase. The macrovoid structure increases after loading 0.1–0.4 wt.% of CNM/PAC (Figure 5a–e). But when the CNM/PAC goes up to 0.5 wt.%, the membrane’s appearance has fewer and smaller macrovoids (Figure 5f). These observations suggest that the addition of nanomaterials significantly influences membrane morphology by increasing the viscosity of the casting solution [42,43]. Additionally, Figure 5g,h illustrates the impact of chemical modification using PFTES at 1–2 wt% concentrations by the dip-coating method. The cross-sectional images of P4 membranes show that the macrovoid structure reduced slightly after coating with 1 wt.% PFTES Figure 5g. When coated with 2 wt.% PFTES, a dense skin layer appears near the top surface, as shown in Figure 5h [44,45].

**Figure 6:**
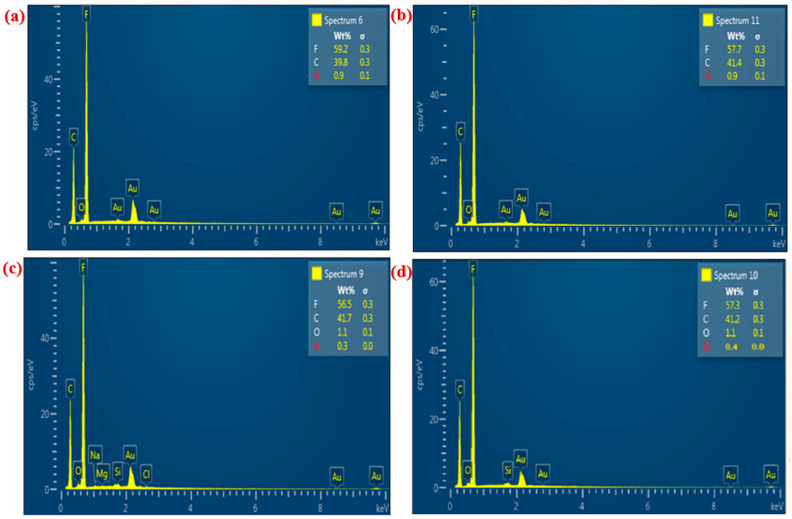
EDX image of (**a**) pristine PVDF (P0), (**b**) 0.4 wt.%CNM/PAC (P4) before and after chemical modification with 1–2 wt.% PFTES (**c**) (1 wt.% FP4), and (**d**) (2 wt.% FP4) membranes.

To evaluate the elemental composition of pristine PVDF membranes before and after the addition of CNM/PAC, Energy Dispersive X-ray Spectroscopy (EDX) was used to analyze the chemical structure of the membranes before and after modification with CNM/PAC and subsequent modification with fluoro-silane (PFTES) solution. EDX spectra were recorded in the binding energy range of 0 to 10 keV, as shown in Figure 6. The elemental analysis of the pristine PVDF membrane (P0) revealed clear peaks corresponding to fluorine (F) and carbon (C), confirming their presence at 59.2 wt.% and 39.8 wt.%, respectively (Figure 6a). For the P4 membrane, which embedded 0.4 wt.% CNM/PAC, an increase in the carbon content to 57.7 wt.% and a slight decrease in fluorine content to 41.4 wt.% was observed, as well as the detection of oxygen (O) at 0.9 wt.% (Figure 6b). This confirms the incorporation of CNM/PAC into the membrane matrix. The presence of PFTES on the surface of the P4 membranes is illustrated by the appearance of silicon (Si) peaks, as shown in Figure 6c,d. At higher PFTES concentrations (2 wt.%), the EDX spectra displayed a slightly higher Si content, confirming increased surface modification (Figure 6c,d). Overall, EDX analysis confirms the successful modification of the PVDF membranes, as indicated by the increase in fluorine content and the detection of Si and O, validating the effective chemical modification with PFTES and the incorporation of CNM/PAC.

### 5.4. Membrane Thickness, Porosity, Pore Size, and LEP

**Figure 7:**
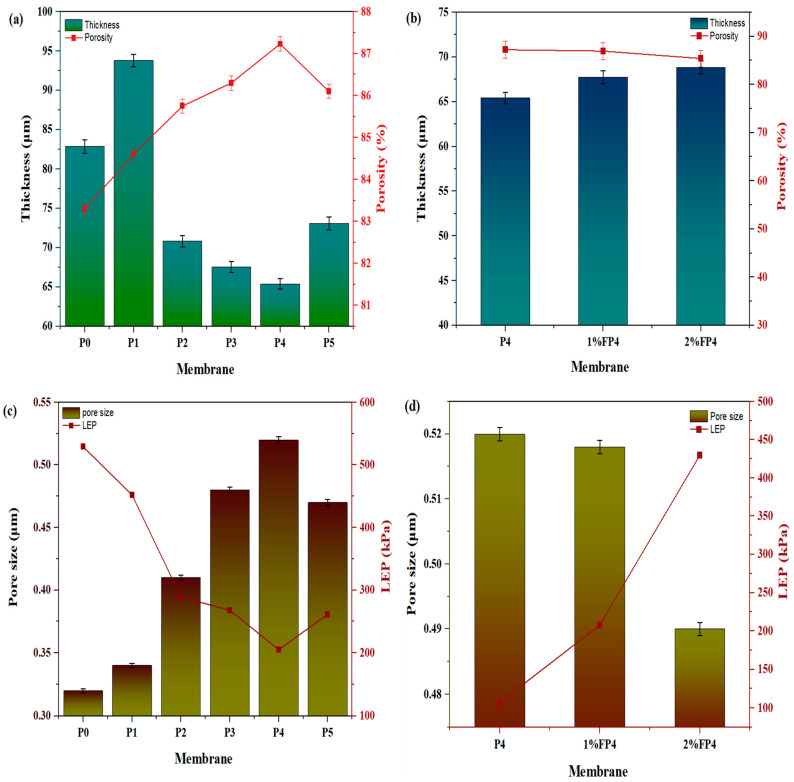
Physical characteristics of membrane before and after modification. (**a**,**b**) Thickness and porosity; (**c**,**d**) pore size.

The properties of the membranes in terms of thickness, porosity, mean pore size, and LEP are presented in Figure 7. Figure 7a-left shows the thickness (82.85 μm) of the pristine PVDF (P0) membranes. Firstly, the thickness rose to 93.79 μm at 0.1% of CNM/PAC (P1) loading. As the concentration of CNM/PAC grew, the thickness reduced. The thickness reduced to 65.41 μm for P4 and then increased to 73.1 μm for P5. Following chemical modification, the thickness of the P4 membrane increased from 65.41 μm to 67.75 μm and 68.73 μm after being coated with 1–2% PFTES. A hydrophobic layer deposited on the membrane surface in proportion to the silane solution concentration (Figure 7b-right) probably causes this rise in thickness.

The porosity of all the prepared membranes was calculated by the dry and wet method (gravimetric analysis). As shown in Figure 7a-right, the porosity of the pristine PVDF (P0) was the lowest, 83.3%, which progressively increased with the incorporation of CNM/PAC, reaching 87.2% for the P4 and slightly reducing to 86.1% for P5. This increase in porosity is probably due to the CNM/PAC dispersion within the polymer matrix, which enhances the rate of exchange between solvent and non-solvent during phase inversion, thus promoting the formation of a more porous structure. Following the chemical modification, the P4 membrane porosity decreased slightly after modification with 1 wt.% and 2 wt.% PFTES. The chemically modified membrane decreased from 87.2% to 86.95% and 85.4% (Figure 7b-right). As the concentration of PFTES solution increased, the modified membrane’s porosity slightly decreased because some small or large pores of the membranes were clogging, resulting in a slight reduction in the water permeate flux.

The mean pore size of all the prepared membranes is shown in Figure 7c. It can be seen that as the CNM/PAC increases, the pore size increases. The pore size increased from 0.32 μm to 0.52 μm as the CNM/PAC concentrations increased from 0.1% (P1) to 0.4 wt.% (P4) and then decreased to 0.47 after loading 0.5 wt.% CNM/PAC (P5). The addition of 0.5 wt.% CNM/PAC may be caused by increasing the viscosity of the casting solution, delaying the rate of exchange between the solvent and non-solvent, and reducing the pore size. In addition, Figure 7c displays the effect of CNM/PAC on the LEP. It can be seen from that figure the LEP decreased as the CNM/PAC went up from 0.1 to 0.4 wt.% due to the inverse relation with pore size. On the other hand, Figure 7d displays the effect of chemical modification on pore size and LEP. After modification with 1 and 2% PFTES, the mean pore size decreased slightly from 0.52 to 0.5 μm and 0.49 μm. This reduction in mean pore size may be due to pore shrinkage caused by the deposition of PFTES on the membrane surface. The decrease in pore size and porosity may cause a reduced permeate flux [19] while increasing the LEP. The increase in LEP post-modification indicates enhanced hydrophobicity and wetting resistance, which are crucial for maintaining membrane performance during long-term MD operation [46]. However, it can be seen that the 2% FP4 membrane shows only a slightly smaller pore size than the P4 membrane but significantly higher LEP than the P4 membrane. This observation was attributed to the slight reduction in pore size of the 2% FP4 membrane compared to the P4 membrane because of the effect of the fluoro-silane solution. At high concentrations of fluoro-silane, the pore size may be partially reduced by the silane solution (PFTES), resulting in a reduction in the pore size, whereas the enhancement in LEP was due to the inverse relation with the pore size and also attributed to the effect of PFTES on the hydrophobicity of the surface. When coating the membrane with 2%PFTES, the contact angle increased from 84° to 117° and the LEP was increased accordingly.

### 5.5. Water Contact Angle

**Figure 8:**
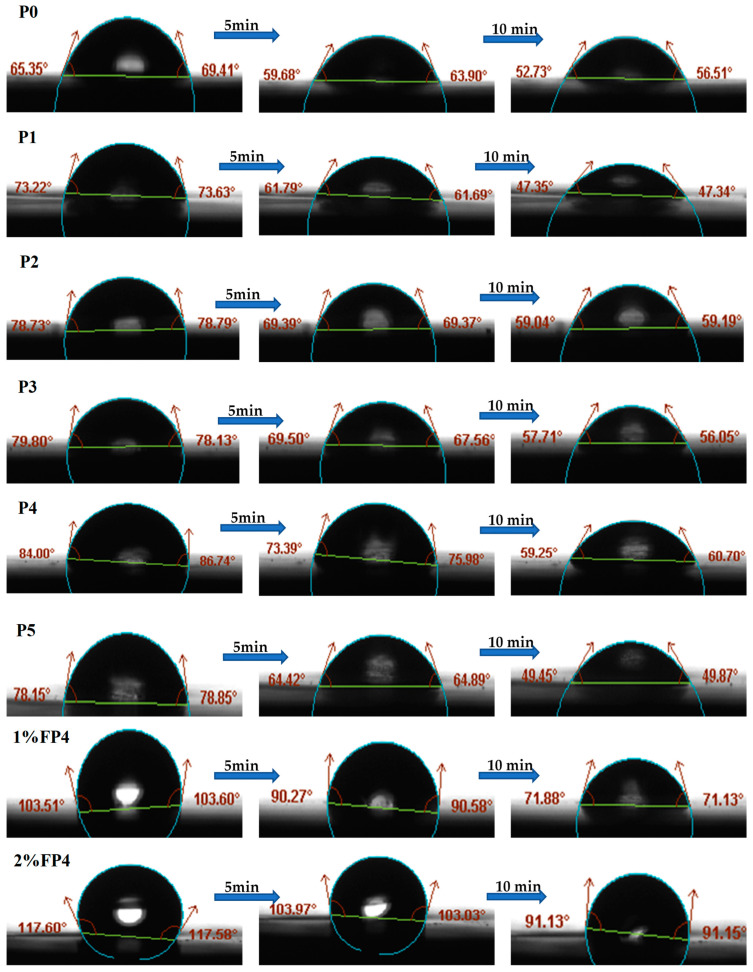
Contact angle with time for P0, P1, P2, P3, P4, P5, and chemical modification of (P4) with 1–2 wt.% PFTES (1 wt.% FP4), (2 wt.% FP4) membranes.

The contact angle plays an important role in evaluating the wettability of the membrane surface, which directly influences its hydrophobicity and efficiency in membrane distillation (MD) [47]. Figure 8 shows that the water contact angle for the pristine PVDF membrane (P0) was 65°. After the addition of 0.1% (P1), 0.2 wt.% (P2), 0.3 wt.% (P3), and 0.4 wt.% (P4) CNM/PAC, the contact angle increased to 73°, 78°, 79°, and 84°, respectively (Figure 8). The result indicates that embedding of CNM/PAC nanomaterials enhances the hydrophobicity of the membrane surface. The loaded nanomaterials increased roughness, leading to micro/nano-scale surface roughness, which in turn traps air pockets and increases the membrane’s hydrophobic character [48,49]. This effect can be attributed to the surface modification achieved through nanomaterial embedding, which promotes the formation of air gaps that reduce liquid–solid contact, thereby increasing the contact angle [35]. However, as the CNM/PAC concentration was further increased to 0.5 wt%(P5), the contact angle decreased to 78°, suggesting that at higher concentrations of CNM/PAC, it may aggregate, potentially reducing the surface roughness and impairing the hydrophobicity [42]. Additionally, the water contact angle slightly decreased over time.

Also, Figure 8 displays the contact angle of the P4 membrane before and after chemical modification with 1 wt.% and 2 wt.% PFTES. It is evident that increasing the fluoro-silane concentration led to a significant increase in the water contact angle. The contact angle rose from 84° (P0) to 111° (1 wt.% FP4) and 117° (2 wt.% FP4). This suggests that the fluoro-silane coating effectively reduced the surface energy of the membrane, rendering it more hydrophobic. The increased contact angle can be explained by the deposition of fluoro-silane molecules, which create a uniform hydrophobic layer on the membrane surface [19]. Additionally, as shown in Figure 8, the contact angle of P4 and the PFTES-coated membranes (1 wt.% FP4 and 2 wt.% FP4) decreased slightly and remained hydrophobic over time, indicating the durability of the hydrophobicity under continuous exposure to water. This stability is critical for maintaining membrane performance during prolonged operation in MD systems.

### 5.6. Atomic Force Microscopy (AFM)

**Figure 9:**
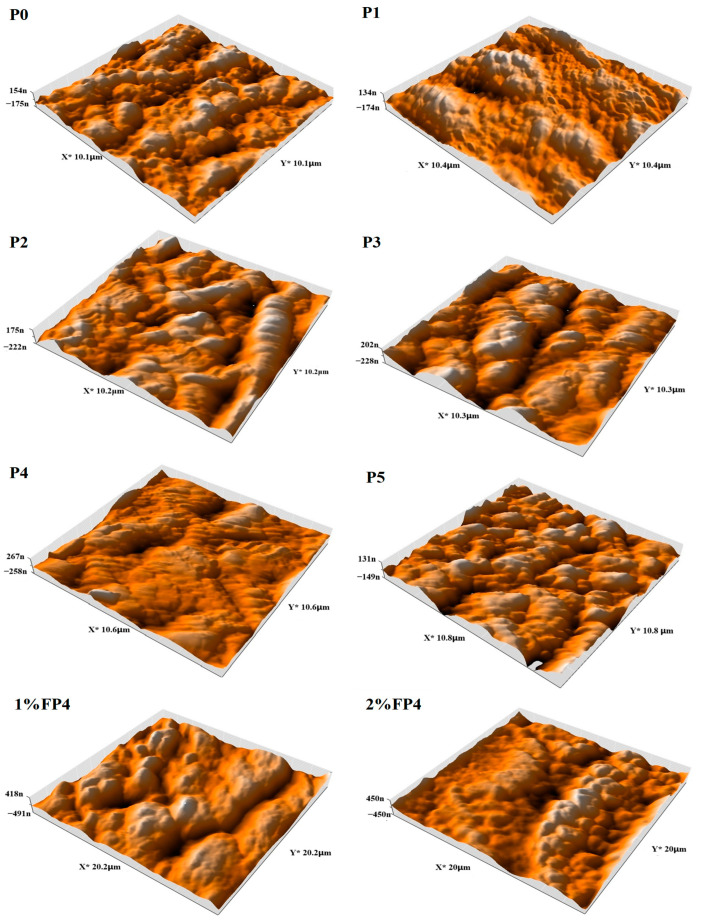
AFM images for P0, P1, P2, P3, P4, P5, and chemical modification of (P4) with 1−2 wt.% PFTES (1% FP4), (2% FP4) membranes.

Since the surface contact angle affects the wetting, it is crucial to evaluate the surface roughness of any membrane to measure its effect on wetting [50,51,52,53]. Figure 9 displays the surface roughness of the PVDF membrane before and after modification with CNM/PAC and chemical modification. The pristine PVDF (P0) membrane had the smoothest surface roughness (Sa = 64.76 nm), as seen in Figure 9. After adding 0.1–0.4 wt.% of CNM/PAC, the roughness of the surface rose to 66.96, 81.53, 93.94, and 95.93 nm and then decreased after loading 0.5 wt.%(P5) (Figure 9). The aggregate of nanomaterials lowers both surface hydrophobicity and roughness [20]. Also, Figure 9 demonstrates the utilization of AFM to test the roughness of the chemically modified P4 membrane. As the concentration of PFTES solution increased, the chemically modified membrane showed a higher surface roughness compared to the original CNM/PAC membrane. The average surface roughness for the P4 (0.4 wt.%) CNM/PAC), (1 wt.% FP4), and (2 wt.% FP4) membranes increased from 95.93 to 156.3 and 175 nm, respectively, as shown in Table 2. In addition to hydrophobicity, the AFM analyzer has been used to determine the porosity and pore size distribution of the prepared membranes. The surface porosity, however, behaves in the opposite way. For P0, P1, P2, P3, P4, and P5, the corresponding surface porosities were found to be 26.64, 26.75, 27.01,27.88, 32.01, and 19.99%. After adding 1 wt.% PFTES to P4 (1 wt.% FP4), the surface porosity increased to 64.83. However, after being treated with 2 wt.% PFTES (2 wt.% FP4) it reduced to 38.97%, but it remains higher than the origin P4 membranes. This increase in surface porosity was due to the large number of maximum pore sizes on the membrane surface. This indicates the increase in the surface porosity after dip-coating [41].

The surface pore size indicates an increase as the concentration of CNM increases. The surface pore size increased from 0.044 μm to 0.087 μm and 0.131 μm for P0, P1, and P4, respectively, then decreased after loading 0.5 wt.% CNM to 0.107 μm. After chemical modification with 1 wt.% PFTES, the pore size of the P4 membrane increased from 0.131 to 0.232 μm and then decreased to 0.142 μm with the saline solution increased up to 2%.

### 5.7. Mechanical Characteristic of Membrane

**Figure 10:**
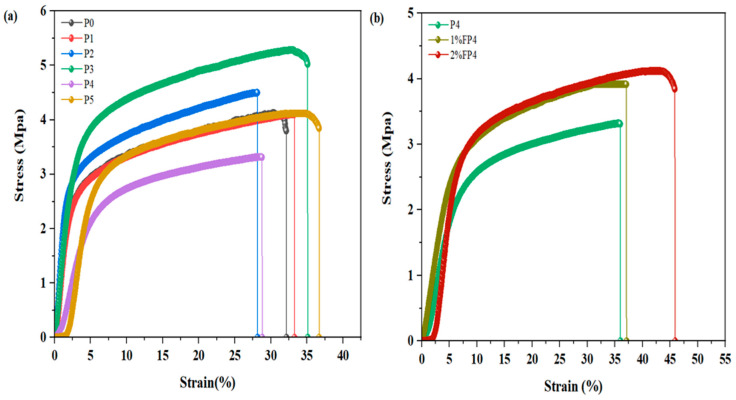
Stress–strain characteristics of (**a**) P0, P1, P2, P3, P4, P5 membrane; (**b**) P4 before and after chemical modification with 1–2 wt.% PFTES (1% FP4, 2% FP4) membranes.

The mechanical characteristics of membranes play an important role in finding their ability to withstand high pressure differences over a long period of VMD [50]. Figure 10a demonstrates the stress–strain properties of membranes with varying CNM/PAC loadings. It is clear that increasing the concentration of CNM/PAC generally resulted in an increase in tensile strength. The pristine PVDF membrane (P0) showed the lowest tensile strength at 3.8 MPa. However, the tensile strength of the modified membranes increased gradually as the CNM/PAC was increased. The tensile stress increased from 3.8 MPa to 4.1 MPa, 4.5 MPa, and 5.025 MPa for P1, P2, and P3, respectively. On the other hand, as a CNM/PAC concentration increased to 0.4 wt.% (P4) and 0.5 wt.% (P5), the tensile strength decreased to 3.312 MPa and 3.845 MPa, respectively. This decline may be attributed to poor dispersion and excessive aggregation of CNM/PAC within the polymer matrix. The aggregation of nanomaterials leads to stress concentration points, which reduce the overall mechanical strength of the membrane [42]. Thus, while CNM/PAC incorporation influences membrane properties, high loading levels must be carefully controlled to avoid compromising mechanical integrity through structural defects and material agglomeration.

Figure 10b displays the tensile stress–strain characteristics of the (P4) membrane after chemical modification with 1 wt.% and 2 wt.% PFTES. The chemically modified membrane of P4 enhanced its mechanical properties upon modification with PFTES. The origin P4 membrane exhibits a tensile strength of 3.8312 MPa, while the chemically modified membrane with 1 and 2% PFTES displays an increase to 3.915 MPa and 4.11 MPa. This enhancement in mechanical properties suggests that the dual modification (incorporating CNM/PAC and PFTES) prevents a significant loss of mechanical strength. The improved tensile strength in 1% FP4 and 2% FP4 makes them more suitable for long-term vacuum membrane distillation (VMD) processes. The increase in tensile strength for the dual-modified membranes, especially when compared to the pristine and solely modified membranes, provides an advantage in terms of mechanical durability, which is critical for MD applications that involve high operating pressure difference and extended usage periods.

## 6. Membrane Performance

### 6.1. Pure Water and Salt Solution

**Figure 11:**
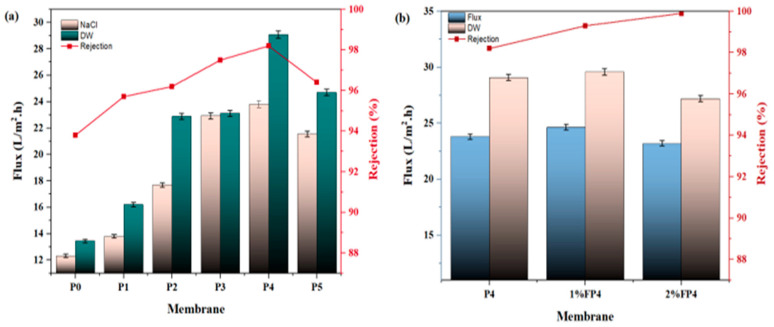
VMD performance for (**a**) P0, P1, P2, P3, P4 and P5 membranes; (**b**) P4 before and after chemical modification with 1–2%PFTES (1% FP4), (2% FP4).

The performance of all prepared membranes in vacuum membrane distillation (VMD) was examined for both distilled water and simulated seawater at 35,000 ppm NaCl under operating conditions of 65 °C feed temperature, 0.6 L/min feed flowrate, and 21 kPa(abs) vacuum pressure. Figure 11a shows the permeate flux through the pristine PVDF (P0) membrane before and after loading different concentrations of CNM/PAC P1 to P5. The results demonstrated that the permeate flux increased by about 11.9%, 43.4%, 94.1%, 99.6%, and 74.7% for P1, P2, P3, and P4, respectively, compared to the pristine PVDF membrane (P0). This enhancement in flux may be due to the addition of CNM/PAC, which improves membrane properties such as porosity and surface hydrophobicity and reduces thickness, offering a larger surface area for evaporation and lower resistance to mass transfer. The highest permeate flux was observed in the P4 membrane (0.4 wt.% CNM/PAC), achieving 23.81 L/m^2^·h. The presence of CNM/PAC also enhances water vapor transfer by acting as an adsorbent, thereby improving both flux and membrane selectivity. This trend suggests that the optimal amount of CNM/PAC leads to enhanced membrane morphology, hydrophobicity, and porosity, which contributes to enhanced flux performance.

Also, Figure 11a demonstrates the salt rejection performance of all the membranes. The pristine PVDF membrane (P0) exhibited a salt rejection rate of 93.8%, indicating the presence of pore wetting and low hydrophobicity. In contrast, the salt rejection rates of P4 exceeded 98.2%, signifying improved membrane hydrophobicity and resistance to pore wetting, which are crucial for effective desalination. Figure 11b displays the effect of varying silane solutions on the performance of the membrane. The figure shows an increase in permeate flux to 24.64 L/m^2^·h upon coating with 1% silane solution as a result of expanding the surface pore size caused by the evaporation of the hexane-silane solution and increased surface porosity. However, this increase was decreased to 23.2 L/m^2^·h when the concentration of silane solution increased to 2 wt% PFTES, as the surface pore size shrank, with the stability of salt rejection above 99.9%.

It is worth mentioning here that the membrane coated with 1 wt.% PFTES had a higher permeate flux and lower rejection than the membranes coated with the 2 wt.% PFTES. This was due to the moderate effect of a coated layer of 1 wt.% PFTES on the physical properties of the membrane surface, such as surface porosity and pore size, caused by the evaporation of the hexane-silane solution, resulting in higher permeate flux and low rejection of salt solution compared to using a high concentration of PFTES (2%) as a coated layer, as shown in Figure 4h.

### 6.2. Effect of Operating Conditions

**Figure 12:**
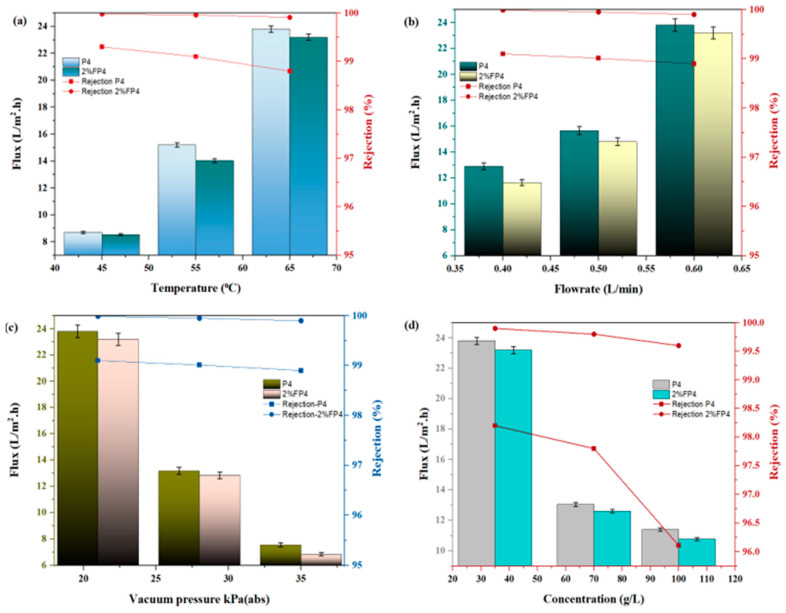
VMD performance for P4 before and after chemical modification (**a**) effect of feed temperature; (**b**) effect of feed flow rate; (**c**) effect of vacuum pressure; (**d**) effect of feed concentration.

The temperature of the feed solution has a direct impact on evaporation rates; it is an important parameter affecting the performance of the membrane in the MD process. This study investigates the effect of feed temperature at 45–65 °C on permeate flux for salt solution at 35 g/L while keeping other parameters constant, such as feed flow rate and vacuum pressure of 0.6 L/min and 21 kPa (abs), respectively. Figure 12a illustrates that an increase in feed temperature enhanced the permeate flux; this is due to increased evaporation of the feed solution at elevated temperatures, which raises the driving force between the two sides of the membrane [51,52,53]. As the temperature increased from 45 °C to 65 °C, the permeate flux for the P4 membrane rose from 8.6 to 23.82 L/m^2^·h. CNM/PAC-loaded membranes showed considerably higher permeate flux. After being chemically modified with 2 wt.% PFTES, the 2% FP4 membranes’ permeate flux rose from 7.53 to 23.2 L/m^2^·h. The reason for this rise in flux is that the permeate side vacuum pressure remained constant, but the partial vapor pressure increased at higher feed temperatures, increasing the driving force for vapor transport. Also, Figure 12a highlights the effect of feed rejection. The results indicate that higher feed temperatures negatively impacted salt rejection due to increased pore wetting. As feed temperature rises, the surface tension decreases, making it easier for the pores to wet, which reduces the membrane’s rejection capacity [54].

Feed flow is a critical parameter in the VMD process due to its impact on temperature gradients across the membrane and its ability to reduce both temperature and concentration polarization phenomena at the membrane surface [55]. Figure 12b shows the variation in permeate flux under different feed flow rates (0.4, 0.5, and 0.6 L/min). The data reveal that the permeate flux increased across the membranes. The permeate flux of P4 increased from 8.9 to 23.82 L/m^2^·h. After PFTES chemical modification, the flux for P4 further increased from 7.82 to 23.2 L/m^2^·h. The observed increase in permeate flux with higher feed flow rates can be attributed to improved Reynolds numbers, which reduce mass and heat transfer resistance by minimizing concentration and temperature polarization effects (i.e., reducing the thickness of the boundary layer) on the feed side, thus enhancing flux performance [54,56,57]. Also, Figure 12b shows the effect of feed flow rate on salt rejection. It was observed that water conductivity increased at higher feed flow rates, likely due to the increased internal pressure that may surpass the liquid entry pressure (LEP) of the membrane, thereby enhancing wettability and reducing salt rejection [58].

Vacuum pressure is a critical parameter that significantly influences both permeate flux and the overall operation of the VMD process [59]. Figure 12c illustrates the effect of vacuum pressure on the permeate flux for a 35 g/L salt solution using PVDF membranes, both before and after chemical modification at various vacuum pressures from 21 to 35 kPa (abs), while other conditions remained constant. In general, the efficiency of the VMD process increased linearly as vacuum pressure decreased. Specifically, the permeate flux of P4 increased from 7.56 to 23.8 L/m^2^·h. Following chemical modification, the permeate flux of 2% FP4 further improved, increasing from 6.8 to 23.2 L/m^2^·h as the vacuum pressure was reduced from 35 to 21 kPa (abs). This rise in permeate flux is mostly due to the improved driving force (transmembrane vapor pressure gradient) between the feed and permeate sides of the membrane, which becomes more pronounced as vacuum pressure decreases, minimizing thermal losses [56,60,61]. Moreover, Figure 12c highlights the effect of vacuum pressure on salt rejection. A higher rejection percentage was observed with decreasing vacuum pressure, likely due to the intensified driving force at lower pressures, which reduces the likelihood of pore wetting and improves separation efficiency [54,62].

Figure 12d shows the effect of varying feed salt concentrations on the permeate flux in VMD for the membranes P4, and 2% FP4. Increasing salt concentration negatively affected permeate flux across all membranes. For instance, the permeate flux of P4 dropped from 23.81 to 11.4 L/m^2^·h whereas the chemically modified membrane with 2 wt.% PFTES (2% FP4) experienced a decline from 23.2 to 10.78 L/m^2^·h as salt concentration increased from 35 to 100 g/L. This reduction in permeate flux with increasing salt concentration is attributable to a decrease in water activity and partial vapor pressure. Additionally, a higher salt concentration exacerbates concentration polarization, which adds resistance to mass transfer and reduces the vapor pressure differential across the membrane [63].

### 6.3. Leaching Nanomaterials and Longtime of Operation

**Figure 13:**
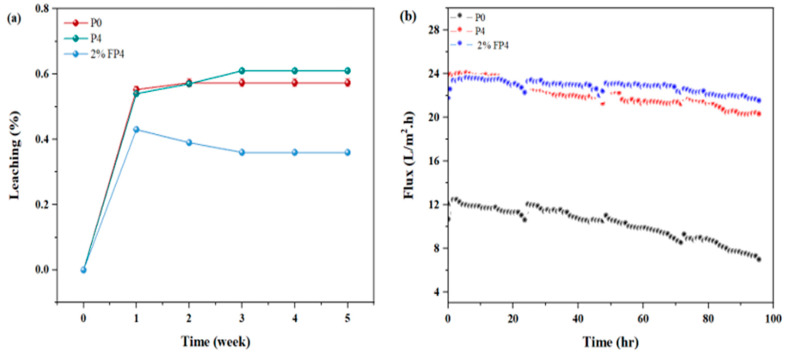
(**a**) Leaching ratio for P0, P4, and 2% FP4 membranes; (**b**) long-term operation test.

The leaching of nanomaterials is one of the challenges that affect membrane performance. Figure 13a displays the leaching test of P0 and CNM/PAC-PVDF(P4) before and after chemical modification with 2 wt.% PFTES (2% FP4). The P4 membrane exhibited an initial leaching ratio of 0.54%, which eventually stabilized at 0.61%. The stability of CNM/PAC within the membrane matrix is crucial for maintaining optimal performance in vacuum membrane distillation (VMD) applications. The chemically modified membrane (2% FP4) demonstrated a higher initial leaching rate of 0.43%, likely due to the release of residual fluoro-silane. However, the leaching rate gradually decreased and stabilized at 0.36%, suggesting improved stability over time. Studies suggest that fluoro-silane, when used as a cross-linking agent, enhances the mechanical properties of the membrane, reducing long-term leaching [64]. Figure 13b illustrates the operational stability of the P0, P4, and 2% FP4 membranes over 96 h. The permeate flux for P0 decreased by approximately 44.7%, while P4 experienced a more moderate decline of 15.5%. In contrast, the chemically modified 2% FP4 membrane exhibited superior stability, with only an 8.4% reduction in flux. This slight decrease indicates that the modified membrane maintained its structural integrity and performance during prolonged operation, preserving its morphological properties.

The evaluation of the chemical stability of the membrane surface prepared by the current work was assessed according to the results of the long-term operation of the membranes shown in Figure 13b. It can be seen that the permeation flow rate for long periods of time of P4 and 2% FP4 membranes was approximately stable for 4 days of long-term operation. This observation indicated that the surface of the membrane was chemically stable. 

Table 3 shows a comparison of PVDF membranes coated with different types of silane solution and used for water desalination by membrane distillation listed in the literature. The comparison presents many properties of the membranes, such as contact angle, operating time, flux, and rejection. The CNM/PAC/PVDF membrane coated with 2% PFTES demonstrates high performance in terms of permeate flux and high removal.

## 7. Conclusions

In this study, we enhanced the hydrophobicity and the salt rejection of the CNM/PAC/PVDF membrane by chemical modification with PFTES at various concentrations. Embedded CNM/PAC nanomaterials improved porosity, pore size, mechanical properties, and hydrophobicity and reduced thickness, which in turn enhanced the permeate flux and salt rejection. The permeate flux for P4 was 23.81 L/m^2^·h, which was 48.2% higher than the pristine PVDF membrane (P0), and the salt rejection was 98.2%. Further chemical modification with 1% PFTES enhanced the permeate flux to 24.6 L/m^2^·h and salt rejection to 99.3% due to the increase in surface pore size and porosity, while chemical modification with 2 wt.% PFTES reduced the permeate flux to 23.2 L/m^2^·h and enhanced the salt rejection and mechanical properties to 99.9% and 4.1 MPa. Also, it can be concluded from the FTIR analysis of the PVDF membrane modified by different amounts of PFTES that the Si-O peak in FTIR corresponded to the covalent bonds between the PFTES and the PVDF membrane surface, which indicated that the surface of the membrane was saturated with PFTES. Furthermore, the long-term stability test revealed a lower degradation (7.76%) after chemical modification with 2 wt.% PFTES with the stability of salt rejection above 99.9%. In order to confirm the stability of chemical modification, leaching tests were conducted over five weeks, and it was found that the 2% FP4 membrane showed a stable leaching rate of 0.36, which confirms the minimal release of nanomaterials after chemical modification. These results highlight the potential of different concentrations of PFTES for membrane performance and salt rejection to improve desalination efficiency over VMD and maintain durability over a long period of operation.

## Figures and Tables

**Table 1:** The composition of CNM/PAC/PVDF membrane.

Sample	PVDF (wt.%)	NMP (wt.%)	CNM/PAC (wt.%)
P0	14	86	--
P1	14	86	0.1
P2	14	86	0.2
P3	14	86	0.3
P4	14	86	0.4
P5	14	86	0.5

**Table 2:** Roughness characteristic of all the prepared membranes.

Membrane Code	Average Surface Roughness (Ra) nm	Root Mean Square (Rq) nm	Maximum Height (Rz) nm
P0	64.76	78.68	455.6
P1	66.96	83.28	499.9
P2	81.53	100.2	641.6
p3	93.94	115.2	706.3
p4	95.93	115.1	600
P5	56.17	69.56	466.2
1% FP4	156.3	196.3	1294
2% FP4	175	212.7	1122

**Table 3:** Some surveys from the literature for fluoro-silane-coated PVDF membranes in MD.

MD Conf.	Polymer	Fluorosilane Type	Contact Angle (°)	Operating Time (h)	Flux (kg/m^2^·h)	Rejection (%)	Ref.
DCMD	PVDF-HFP	perfluorodecyldimethylchlorosilane (FAS-17)	138	/	19	99.9	[65]
DCMD	PVDF	Perfluorodecyltriethoxysilane (PFDES)	162	/	/	99.9	[66]
AGMD	PVDF	Heneicosafluorododecyltrichlorosilane (FAS-21)	>150	/	19.09	99.9	[41]
DCMD	PVDF	methyl trichloroalkyl silane (MTCS) perfluorooctane trichlorosilane silanes (PTCS)	112 116	/	/	/	[19]
VMD	PVDF	Perfluorodecyltriethoxysilane (PFTES)	117	96	23.2	99.9	This work

## Data Availability

Dataset available on request from the authors.

## References

[1] Patel R.V., Chaubey S., Yadav A., Shahi V.K. (2024). Chemical grafting of hydrophobic functional groups on polyvinylidene fluoride side chain for vacuum membrane distillation applications. J. Environ. Chem. Eng..

[2] Asadolahi M., Fashandi H., Zamani H. (2024). Disclosing the pivotal role of Poly(vinylidene fluoride) omniphobic membrane surface morphology in performance of photothermal vacuum membrane distillation. Chem. Eng. J..

[3] Zhang N., Zhang J., Gao C., Yuan S., Wang Z. (2025). Emerging advanced membranes for removal of volatile organic compounds during membrane distillation. Desalination.

[4] Sun B., Wu M., Zhen H., Jia Y., Li P., Yuan Z., Li X., He G., Jiang X. (2024). Tailored PVDF membrane with coordinated interfacial nano/micro-structure for enhanced membrane distillation. Desalination.

[5] Julian H., Nurgirisia N., Qiu G., Ting Y.-P., Wenten I.G. (2022). Membrane distillation for wastewater treatment: Current trends, challenges and prospects of dense membrane distillation. J. Water Process Eng..

[6] Chin J.Y., Ahmad A.L., Low S.C. (2020). Anti-Wetting Membrane Distillation to Treat High Salinity Wastewater: Review. J. Membr. Sci. Res..

[7] Asadolahi M., Fashandi H., Ghodsi A. (2024). One-step development of photothermal omniphobic membrane for vacuum membrane distillation towards sustainable water desalination using solar energy. Chem. Eng. J..

[8] Samadi A., Ni T., Fontananova E., Tang G., Shon H., Zhao S. (2023). Engineering antiwetting hydrophobic surfaces for membrane distillation: A review. Desalination.

[9] Pangarkar B.L., Deshmukh S.K., Sapkal V.S., Sapkal R.S. (2016). Review of membrane distillation process for water purification. Desalin. Water Treat..

[10] Ahmadi H., Ziapour B.M., Ghaebi H., Nematollahzadeh A. (2024). Optimization of vacuum membrane distillation and advanced design of compact solar water heaters with heat recovery. J. Water Process Eng..

[11] Rashid K.T., Rahman S.B.A., Alsalhy Q.F. (2016). Optimum Operating Parameters for Hollow Fiber Membranes in Direct Contact Membrane Distillation. Arab. J. Sci. Eng..

[12] Abid M.B., Wahab R.A., Salam M.A., Gzara L., Moujdin I.A. (2023). Desalination technologies, membrane distillation, and electrospinning, an overview. Heliyon.

[13] Francis L., Ahmed F.E., Hilal N. (2022). Advances in Membrane Distillation Module Configurations. Membranes.

[14] Alsebaeai M.K., Ahmad A.L. (2020). Membrane distillation: Progress in the improvement of dedicated membranes for enhanced hydrophobicity and desalination performance. J. Ind. Eng. Chem..

[15] Suga Y., Takagi R., Matsuyama H. (2021). Effect of the characteristic properties of membrane on long-term stability in the vacuum membrane distillation process. Membranes.

[16] An Y., Li F., Wang H., Zhang X., Di Y., Wang L., Yan Z., Wang W., Lu J., Liu M. (2025). Preparation and properties of cellulose triacetate (CTA)-PVDF composite membrane with hydrophobic/hydrophilic properties for membrane distillation. Appl. Surf. Sci..

[17] Zheng R., Chen Y., Wang J., Song J., Li X.-M., He T. (2018). Preparation of omniphobic PVDF membrane with hierarchical structure for treating saline oily wastewater using direct contact membrane distillation. J. Memb. Sci..

[18] Schnittger J., McCutcheon J., Hoyer T., Weyd M., Fischer G., Puhlfürß P., Halisch M., Voigt I., Lerch A. (2021). Hydrophobic ceramic membranes in MD processes—Impact of material selection and layer characteristics. J. Membr. Sci..

[19] Jin Q., Zhang X., Li F., Zhao X. (2023). Hydrophobic modification of a PVDF hollow fiber membrane by plasma activation and silane grafting for membrane distillation. Water Sci. Technol..

[20] Swaminathan M., Swaminathan G. (2024). An Insight into Enhanced Membrane Hydrophobicity Using Silane Functionalization for Effective Membrane Distillation. Sep. Purif. Rev..

[21] Van Tran T.T., Nguyen C.H., Lin W.-C., Juang R.-S. (2021). Improved stability of a supported liquid membrane process via hydrophobic modification of PVDF support by plasma activation and chemical vapor deposition. Sep. Purif. Technol..

[22] Arkles B. (2006). Hydrophobicity, hydrophilicity and silanes. Paint. Coat. Ind..

[23] Arkles B. (1977). Tailoring Surfaces with Silanes. Chemtech.

[24] Aljumaily M.M., Alsaadi M.A., Das R., Abd Hamid S.B., Hashim N.A., AlOmar M.K., Alayan H.M., Novikov M., Alsalhy Q.F., Hashim M.A. (2018). Optimization of the synthesis of superhydrophobic carbon nanomaterials by chemical vapor deposition. Sci. Rep..

[25] Almajras Q.A., Hassan A.K., Al-Juboori R.A., Alsalhy Q.F. (2025). Green and sustainable biosynthesis of hybrid iron/palladium nanoparticles functionalized PES membranes for dye removal. Desalin. Water Treat..

[26] Tai B., Ma X. (2019). Superhydrophobic Surface Modification of Carbon Nanotubes for Polymer Membrane in Direct Contact Membrane Distillation. Master’s Thesis.

[27] Mpala T.J., Richards H., Etale A., Mahlangu O.T., Nthunya L.N. (2023). Carbon nanotubes and silver nanoparticles modification of PVDF membranes for improved seawater desalination in direct contact membrane distillation. Front. Membr. Sci. Technol..

[28] Hussein Al-Timimi D.A., Alsalhy Q.F., AbdulRazak A.A. (2023). Polyethersulfone/amine grafted silica nanoparticles mixed matrix membrane: A comparative study for mebeverine hydrochloride wastewater treatment. Alex. Eng. J..

[29] Ibraheem B.M., AlAani S., Alsalhy Q.F., Nemeth Z., Hernadi K. (2023). Improving surface characteristics and operational parameters of polyvinyl chloride ultrafiltration membranes: How to manipulate the performance of thin film composite forward osmosis membranes?. Desalination.

[30] Francis L., Hilal N. (2022). Electrosprayed CNTs on Electrospun PVDF-Co-HFP Membrane for Robust Membrane Distillation. Nanomaterials.

[31] Saffarini R., Arafat H., Thomas R. Influence of Pore Structure on Membrane Wettability in Membrane. Proceedings of the Sixth Jordan International Chemical Engineering Conference.

[32] Racz G., Kerker S., Schmitz O., Schnabel B., Kovacs Z., Vatai G., Ebrahimi M., Czermak P. (2015). Experimental determination of liquid entry pressure (LEP) in vacuum membrane distillation for oily wastewaters. Membr. Water Treat..

[33] Yang C., Peng X., Zhao Y., Wang X., Cheng L., Wang F., Li Y., Li P. (2019). Experimental study on VMD and its performance comparison with AGMD for treating copper-containing solution. Chem. Eng. Sci..

[34] Rao L., You X., Chen B., Shen L., Xu Y., Zhang M., Hong H., Li R., Lin H. (2022). A novel composite membrane for simultaneous separation and catalytic degradation of oil/water emulsion with high performance. Chemosphere.

[35] Aljumaily M.M., Alsaadi M.A., Hashim N.A., Alsalhy Q.F., Das R., Mjalli F.S., Awanis Hashim N., Alsalhy Q.F., Das R., Mjalli F.S. (2019). Embedded high-hydrophobic CNMs prepared by CVD technique with PVDF-co-HFP membrane for application in water desalination by DCMD. Desalin. Water Treat..

[36] Tian X., Jiang X. (2008). Poly(vinylidene fluoride-co-hexafluoropropene) (PVDF-HFP) membranes for ethyl acetate removal from water. J. Hazard. Mater..

[37] Silva T.L.S., Morales-Torres S., Figueiredo J.L., Silva A.M.T. (2015). Multi-walled carbon nanotubes/PVDF blended membranes with sponge-and finger-like pores for direct contact membrane distillation. Desalination.

[38] Avilés F., Cauich-Rodríguez J.V., Moo-Tah L., May-Pat A., Vargas-Coronado R. (2009). Evaluation of mild acid oxidation treatments for MWCNT functionalization. Carbon.

[39] Wu T., Zhou B., Zhu T., Shi J., Xu Z., Hu C., Wang J. (2014). Facile and low-cost approach towards a PVDF ultrafiltration membrane with enhanced hydrophilicity and antifouling performance via graphene oxide/water-bath coagulation. RSC Adv..

[40] Khayet M., Cojocaru C., García-Payo M.C. (2010). Experimental design and optimization of asymmetric flat-sheet membranes prepared for direct contact membrane distillation. J. Memb. Sci..

[41] Baig U., Azeem M.A., Lawal D.U., Al Abdulgader H., Baroud T.N. (2022). Facile and scalable fabrication of superhydrophobic PVDF membrane for the desalination of highly saline water using air-gap membrane distillation system. J. Environ. Chem. Eng..

[42] Zhou R., Rana D., Matsuura T., Lan C.Q. (2019). Effects of multi-walled carbon nanotubes (MWCNTs) and integrated MWCNTs/SiO_2_ nano-additives on PVDF polymeric membranes for vacuum membrane distillation. Sep. Purif. Technol..

[43] El-Zanati E., Taha E., Ettouney R., El-Rifai M. (2022). Functionalized CNT/PVDF Blended Flat Sheet Membrane for Water Desalination via Vacuum Membrane Distillation, Fabrication and Characterization. Water Energy Food Environ. J. Int. J..

[44] Jiménez-Robles R., Moreno-Torralbo B.M., Badia J.D., Martínez-Soria V., Izquierdo M. (2022). Flat PVDF Membrane with Enhanced Hydrophobicity through Alkali Activation and Organofluorosilanisation for Dissolved Methane Recovery. Membranes.

[45] Jiménez-Robles R., Izquierdo M., Martínez-Soria V., Martí L., Monleón A., Badia J.D. (2023). Stability of Superhydrophobicity and Structure of PVDF Membranes Treated by Vacuum Oxygen Plasma and Organofluorosilanisation. Membranes.

[46] Chimanlal I., Nthunya L.N., Mahlangu O.T., Kirkebæk B., Ali A., Quist-Jensen C.A., Richards H. (2023). Nanoparticle-Enhanced PVDF Flat-Sheet Membranes for Seawater Desalination in Direct Contact Membrane Distillation. Membranes.

[47] Abdelrasoul A. (2020). Advances in Membrane Technologies.

[48] Madaeni S.S., Zinadini S., Vatanpour V. (2013). Preparation of superhydrophobic nanofiltration membrane by embedding multiwalled carbon nanotube and polydimethylsiloxane in pores of microfiltration membrane. Sep. Purif. Technol..

[49] Aljumaily M.M., Alayan H.M., Mohammed A.A., Alsaadi M.A., Alsalhy Q.F., Figoli A., Criscuoli A. (2022). The influence of coating super-hydrophobic carbon nanomaterials on the performance of membrane distillation. Appl. Water Sci..

[50] Aljumaily M.M., Alsaadi M.A., Hashim N.A., Alsalhy Q.F., Mjalli F.S., Atieh M.A., Al-Harrasi A. (2018). PVDF-co-HFP/superhydrophobic acetylene-based nanocarbon hybrid membrane for seawater desalination via DCMD. Chem. Eng. Res. Des..

[51] Devi S., Ray P., Singh K., Singh P.S. (2014). Preparation and characterization of highly micro-porous PVDF membranes for desalination of saline water through vacuum membrane distillation. Desalination.

[52] Pagliero M., Comite A., Soda O., Costa C. (2021). Effect of support on PVDF membranes for distillation process. J. Memb. Sci..

[53] Liu J., Albdoor A.K., Lin W., Hai F.I., Ma Z. (2022). Membrane fouling in direct contact membrane distillation for liquid desiccant regeneration: Effects of feed temperature and flow velocity. J. Memb. Sci..

[54] Chen L., Xu P., Wang H. (2020). Interplay of the Factors Affecting Water Flux and Salt Rejection in Membrane Distillation: A State-of-the-Art Critical Review. Water.

[55] Xu J., Singh Y.B., Amy G.L., Ghaffour N. (2016). Effect of operating parameters and membrane characteristics on air gap membrane distillation performance for the treatment of highly saline water. J. Memb. Sci..

[56] Iqbal F., Asif M., Jafry A.T., Bibi W. (2024). Experimental Investigation of Vacuum Membrane Distillation (VMD) Performance Based on Operational Parameters for Clean Water Production. MATEC Web Conf..

[57] Pangarkar B.L., Sane M.G., Parjane S.B., Abhang R.M., Guddad M. (2010). The heat and mass transfer phenomena in vacuum membrane distillation for desalination. World Acad. Sci. Eng. Technol..

[58] Anvari A., Azimi Yancheshme A., Kekre K.M., Ronen A. (2020). State-of-the-art methods for overcoming temperature polarization in membrane distillation process: A review. J. Memb. Sci..

[59] Abu-Zeid M.A.E.R., Zhang Y., Dong H., Zhang L., Chen H.L., Hou L. (2015). A comprehensive review of vacuum membrane distillation technique. Desalination.

[60] Hussain Mana T., Alam J., Shukla A.K., Alkhudhiri A., Mohammed A.N., Alhoshan M. (2024). Performance investigation of poly(vinylidene fluoride-cohexafluoropropylene) membranes containing SiO_2_ nanoparticles in a newly designed single vacuum membrane distillation system. Water Environ. Res..

[61] Madupathi M.M., Srishti S., Fatima S., Sridhar S. (2024). Sea and brackish water desalination through a novel PVDF-PTFE composite hydrophobic membrane by vacuum membrane distillation. Discov. Chem. Eng..

[62] Kim H., Yun T., Hong S., Lee S. (2021). Experimental and theoretical investigation of a high performance PTFE membrane for vacuum-membrane distillation. J. Memb. Sci..

[63] Giraldo-Mejía H., Quintero Y.M., Mery F., Rodriguez F., Curcio E., Estay H., Garcia A. (2023). Plasma-grafting surface modifications to enhance membrane hydrophobicity for brine membrane distillation. Desalination.

[64] Ji J., Li H., Wang W., Li J., Zhang W., Li K., Yang T., Jin W., Tang Y., Li W. (2024). Silane-crosslinked polybenzimidazole with different hydroxyl content for high-temperature proton exchange membrane. J. Memb. Sci..

[65] An X., Liu Z., Hu Y. (2018). Amphiphobic surface modification of electrospun nanofibrous membranes for anti-wetting performance in membrane distillation. Desalination.

[66] Li B., Yun Y., Wang M., Li C., Yang W., Li J., Liu G. (2021). Superhydrophobic polymer membrane coated by mineralized β-FeOOH nanorods for direct contact membrane distillation. Desalination.

